# Molecular techniques for detecting and typing of bacteria, advantages and application to foodborne pathogens isolated from ducks

**DOI:** 10.1007/s13205-012-0074-4

**Published:** 2012-06-24

**Authors:** Frederick Adzitey, Nurul Huda, Gulam Rusul Rahmat Ali

**Affiliations:** 1 Food Technology Division, School of Industrial Technology, Universiti Sains Malaysia, 11800 Pulau Pinang, Malaysia; 2 Animal Science Department, University for Development Studies, Box TL 1882, Tamale, Ghana

**Keywords:** Ducks, Foodborne pathogens, Molecular techniques, Surveillance studies

## Abstract

In recent times, several foodborne pathogens have become important and a threat to public health. Surveillance studies have provided data and a better understanding into the existence and spread of foodborne pathogens. The application of molecular techniques for detecting and typing of foodborne pathogens in surveillance studies provide reliable epidemiological data for tracing the source of human infections. A wide range of molecular techniques (including pulsed field gel electrophoresis, multilocus sequence typing, random amplified polymorphism deoxyribonucleic acid, repetitive extragenic palindromic, deoxyribonucleic acid sequencing, multiplex polymerase chain reaction and many more) have been used for detecting, speciating, typing, classifying and/or characterizing foodborne pathogens of great significance to humans. Farm animals including chickens, cattle, sheep, goats and pigs, and others (such as domestic and wild animals) have been reported to be primary reservoirs for foodborne pathogens. The consumption of contaminated poultry meats or products has been considered to be the leading source of human foodborne infections. Ducks like other farm animals are important source of foodborne pathogens and have been implicated in some human foodborne illnesses and deaths. Nonetheless, few studies have been conducted to explore the potential of ducks in causing foodborne outbreaks, diseases and its consequences. This review highlights some common molecular techniques, their advantages and those that have been applied to pathogens isolated from ducks and their related sources.

## Introduction

Foodborne pathogens are increasingly being studied due to their ability to change and to adapt to different environmental and surviving conditions. The ability of these pathogens to mutate has contributed to their adaptability and survival under a wide range of conditions. The presence of certain antibodies, virulent genes and/or other complex defensive mechanisms produced by foodborne pathogens also contributes to their adaptability and survival under various environmental conditions. The survival of foodborne pathogens under a variety of environmental conditions warrants the development and use of efficient and reliable isolation, detection, differentiation, classification and/or typing techniques for their surveillance (Adzitey and Nurul 2011; Adzitey et al. 2011). Surveillance studies provide epidemiological data for tracing the source of infection for clinical and treatment purposes. Furthermore, surveillance studies provide data that help to reduce the emergence and colonization of foodborne pathogens, and to adapt appropriate strategies to prevent and control the spread of foodborne.

A variety of foodborne pathogens have been isolated (through surveillance studies) from different foodstuffs, animals, plants and environmental samples that have been implicated in foodborne illnesses, diseases, poisonings and/or intoxications which occurred either sporadically or through outbreaks. In particular, the handling and consumption of contaminated raw poultry meats and products have been implicated in most cases (Centers for Disease Control and Prevention (CDC) 2003; Humphrey et al. 2007; European Food Safety Authority (EFSA) 2008; Frederick and Huda 2011). Duck meats and eggs have also been implicated in a number of outbreaks. For example, an outbreak of *Salmonella* Typhimurium definitive phage type (DT) 8 was associated with duck eggs and products, and was responsible for the hospitalisation of two people and the death of one (Clarke 2010). Contact with young birds and ducklings in a nursery school has been linked to outbreak of *Salmonella* infection (Merritt and Herlihy 2003). Salmonellosis has also been associated with chicks and ducklings (Morbidity and Mortality Weekly Report (MMWR) 2000).

Effective surveillance of foodborne pathogens can be achieved through a combination of the conventional and several polymerase chain reactions (PCR)-based techniques (Loncarevic et al. 2008; Aurora et al. 2009; Adzitey and Corry 2011). The conventional or cultural standard methods appear to have been used since the inception of microbiological sampling (Adzitey and Huda 2010; Adzitey and Nurul 2011). These methods mainly involve enrichment (pre-enrichment and/or selective enrichment) followed by plating onto selective agar or by plating directly onto selective agar without enrichment, and confirmation of presumptive bacteria colonies by biochemical tests (Corry et al. 2003; Adzitey et al. 2011). They are widely used and have the advantage that, they are cheaper, detect only viable bacteria, and yield isolates that can further be characterised and studied (Engberg et al. 2000; Adzitey and Nurul 2011). However, they are laborious, relatively slow and less efficient (Keramas et al. 2004; Myint et al. 2006). Molecular techniques have also been widely used in surveillance, mutation and other genetic studies of foodborne pathogens to increase our understanding into the primary source of foodborne pathogens, source of infection and genetic diversity. Molecular techniques have the advantage that, they are rapid, less laborious, and more sensitive, specific and efficient compared to the conventional method (Magistrado et al. 2001; Keramas et al. 2004). Nonetheless, certain components/compounds in foods such as fats, lipids and salts, enrichment media or DNA extraction solution can inhibit the sensitivity of polymerase chain reaction (PCR)-based methods (Rossen et al. 1992; Wilson, 1997).

The purpose of this paper is to highlight some commonly available molecular techniques, their advantages and usage to detect, characterize and/or to type foodborne pathogens isolated from ducks and duck-related samples.

## Detection methods using polymerase chain reaction (PCR)-based assays

Polymerase chain reaction (PCR) is an in situ DNA replication process that allows for the exponential amplification of target DNA in the presence of synthetic oligonucleotide primers and a thermostable DNA polymerase (Farber 1996; Wang et al. 2000). A wide range of different concentrations or units of DNA templates (5–25 ng), Taq DNA polymerase (0.6–1.25 U), primers (0.11–10 μM), and temperature cycles (45–95.8 °C and 30–40 cycles) have been employed to detect or confirm bacteria isolated from ducks (Boonmar et al. 2007; Rahimi et al. 2011; Su et al. 2011; Adzitey et al. 2012a). Other components of a PCR reaction such as deoxyribonucleotide triphosphates (dNTPs), magnesium (Mg^2+^) and buffer solutions have been used in different concentrations to increase detection limits. A PCR process may involve the use of one primer (single PCR) or multiple primers (multiplex PCR) to detect bacterial isolates (Table 1). Other forms of PCR are real-time PCR, nested PCR, reverse-transcription PCR and many more.

**Table 1:** Molecular methods applied to identify bacteria isolated from ducks and their related samples

Identification method	Purpose	Sample type	Species or serovars	Target gene (s)	References
Single PCR	To identify *Campylobacter* spp.	Mallard duck	*C. jejuni*	NA	Magistrado et al. (2001)
Single PCR	To identify *Campylobacter* spp.	Duck faeces and environmental waters contaminated by duck droppings	*C. jejuni, C. coli*	NA	Abulreesh et al. (2010)
Single PCR	To confirm the identity of *Campylobacter* spp.	Duck meat	*C. jejuni, C. coli*	16S rRNA, *mapA, ceuE*	Rahimi et al. (2011)
Single PCR	To amplify the 16S rRNA of *Campylobacter* spp. prior to sequencing	Caeca	*C. lari*	16S rRNA	Adzitey et al. (2012a)
Multiplex PCR	To speciate *Campylobacter* spp.	Caeca, intestines, cloacal, wash water, floor swab	*C. jejuni, C. coli*	*hipO, glyA, glyA, glyA, sapB2*	Adzitey et al. (2012a)
Multiplex PCR	Compared the detection of *Campylobacter* spp. from duck meat and intestines using multiplex PCR and convention method	Duck meat and intestine	*C. jejuni, C. coli*	NA	Boonmar et al. (2007)
Multiplex PCR	To detect *Salmonella* isolates from duck hatcheries	Duck hatcheries	*S*. Potsdam, *S.* Montevide, *S.* Albany	*invA, wzx, tyv, fliC, fljB*	Su et al. (2011)

*NA* not available

Polymerase chain reaction assays have been routinely used for rapid detection, identification and differentiation of foodborne pathogens. They have been used in areas such as DNA cloning, diagnosis of hereditary and infectious diseases, identification of genetic fingerprints, and detection and diagnosis of infectious diseases. Polymerase chain reaction technique plays an important role in the identification of typical bacterial strains that exist in viable but nonculturable coccoid forms (e.g. *Campylobacter* spp.) which are often missed by the conventional method (Magistrado et al. 2001). The use of PCR also avoids situations where phenotypic characteristics are ambiguous and wrongly interpreted, for instance the occurrence of hippurate negative *C. jejuni* strains (Adzitey and Corry, 2011). However, some PCR’s may not be suitable for processed and certain foods because amplification can be obtained from DNA originating from both viable and non-viable cells (Sails et al. 1998; Wang et al. 2000). The technique can be expensive and its sensitivity and performance can be inhibited by components of enrichment broth and DNA extraction solution, concentration of the PCR mixtures (primers, DNA templates, dNTP’s and Mg^2+^), and temperature and cycling conditions (Rossen et al. 1992; Wilson 1997; Wassenaar and Newell 2000). Table 1 shows commonly available molecular techniques that have been applied to identify bacteria isolated from ducks and their related samples; while Table 2 summarizes the advantages and disadvantages of some commonly available molecular techniques for identifying foodborne pathogens.

**Table 2:** Advantages and disadvantages of some commonly available molecular techniques for identifying foodborne pathogens

Identification method	Advantages	Disadvantages	References
Single PCR^a^	Provides a more accurate, sensitive and rapid detection of single bacteria or genes	Does not produce isolates that can further be characterized, components in foods can interfere with PCR performance and give misleading results, PCR conditions must be optimized for better performance	Sails et al. (1998); Wang et al. (2000); Abulreesh et al. (2006)
Multiplex PCR^a^	Reduces cost, limits sample volumes and allows rapid detection of multiple bacteria	Primer design is critical, primers may interfere with each other leaving some genes and bacteria undetected	Elnifro et al. 2000; Shi et al. (2010)
Real-time PCR^b^	Shortens detection time, detect and quantify bacteria in real time, and high sensitivity, specificity and reproducibility	Require expensive equipment and reagents, setting up requires high technical skills	Heid et al. (1996); Wong and Medrano (2005); Shi et al. (2010)
Reverse-transcription PCR^b^	Can detect only viable cells of pathogens	Much skill is required to handle unstable RNA for pathogen detection	Sails et al. (1998); Sharma (2006); Shi et al. (2010)
Nested PCR^b^	Has improved sensitivity and specificity than the conventional PCR method	Contamination level can be high probably from the laboratory environment	Picken et al. (1997)

^a^Applied to duck bacterial isolates

^b^Their applications to duck bacterial isolates are unavailable or yet to be published

### Single polymerase chain reaction

This is a polymerase chain reaction (PCR) involving the use of a single primer set (which targets a specific gene) to detect an organism. The primer set can be designed for specific species and can detect the target organism in the presence of others. This kind of PCR can be applied to rapidly detect or identify bacteria directly from a sample (food, water, clinical or environmental) with or without pre-enrichment. However, direct detection of foodborne pathogens by PCR assays in the environment or in sample of turbid nature can result in the detection of DNA in dead cells and give false negative results (Josefsen et al. 2004; Abulreesh et al. 2006). Enrichment before PCR detection and/or the application of fluorescence in situ hybridization (FISH) techniques have been suggested to curb this situation (Lehtola et al. 2005; Abulreesh et al. 2006). Single PCR can also be applied to confirm bacteria isolates picked directly from agar plates. In recent times, PCR using universal or specific primers have been used to initially amplify the 16S rRNA genes of bacteria before being sequenced to help in the identification of unknown or novel bacteria species. Magistrado et al. (2001) reported that PCR accurately identified one *C. jejuni* isolate from Philippine Mallard duck. They also showed that PCR can be used to directly detect *Campylobacter* spp., in the presence of other contaminating bacteria and can be enhanced by prior enrichment-plating procedure. Abulreesh et al. (2010) used PCR to identify *Campylobacter* spp. recovered from duck faeces and environmental waters that were contaminated with duck droppings. Rahimi et al. (2011) used PCR to confirm the identity of 39 *Campylobacter* spp., isolated from 110 duck meat samples using the conventional bacteriological method in Iran. Adzitey et al. (2012b) used PCR to amplify the 16S rRNA of *Campylobacter* spp., prior to sequencing for species identification.

### Multiplex polymerase chain reaction

This is a modification of polymerase chain reaction that uses multiple primers within a single PCR mixture to detect, identify and/or differentiate bacteria. Thus, in multiplex PCR more than one target sequences are amplified in a reaction to produce amplicons of varying sizes specific for different DNA sequences. Although multiplex PCR reduces cost, limits volume of samples and allows for the rapid detection of multiple bacteria species, strains and so on, primer design is critical in the development of multiplex PCR (Shi et al. 2010). All primers need to have close annealing temperature, the amplicons must be markedly different in sizes and multiple primers may interfere with each other during the amplification process (Elnifro et al. 2000; Shi et al. 2010). Boonmar et al. (2007) compared the detection of *Campylobacter* species in duck meat and intestines in Nakhon Pathom Province, Thailand using the standard culture method (SCM) and multiplex PCR. They found 20 % (21 *C. jejuni* and 7 *C. coli* strains) and 31 % (34 *C. jejuni* and 10 *C. coli*) positive samples for SCM and multiplex PCR, respectively. Adzitey et al. (2012a) employed a multiplex PCR to differentiate between *Campylobacter* species isolated from ducks and their environs in Penang, Malaysia. They identified 113 *C. jejuni* strains and 22 *C. coli* strains using multiplex PCR. The multiplex PCR they employed was unable to identify three *C. lari* strains which were identified by sequencing. Su et al. (2011) used four multiplex PCR assays to detect *Salmonella* isolates from duck hatcheries.

### Other polymerase chain reaction assays

These encompass commonly available modified PCR techniques that are yet to be applied to foodborne pathogens isolated from ducks. They include real-time PCR, nested PCR and reverse-transcription PCR. Real-time PCR is a polymerase chain reaction process in which the target DNA is amplified and quantified simultaneously within a reaction. Real-time PCR employs specific primer set, one or two probes and/or fluorescent dye to improve detection signals (Rensen et al. 2006; Dhanasekaran et al. 2010; Shi et al. 2010). In real-time PCR, the amplified DNA is detected in real time as the reaction progresses instead of at the reaction end. Real-time PCR shortens detection time compared to standard PCR and can determine the absolute or relative number of bacteria in various samples (Heid et al. 1996; Shi et al. 2010). Furthermore, there is no post-PCR processing of products, leads to high throughput and reduces the risk of amplicon contamination by laboratory environments (Heid et al. 1996; Wong and Medrano 2005; Shi et al. 2010). However, equipment and reagent costs are high for real-time PCR (Wong and Medrano 2005). In reverse-transcription PCR, RNA is used as the initial template instead of DNA. Reverse transcriptase is used to reverse transcribed the target RNA into its DNA complement (cDNA) and amplified using PCR (Sharma 2006). Reverse-transcription PCR is useful in detecting only viable cells of pathogens; however RNA is unstable requiring much skill during handling and quantification for pathogen detection (Sails et al. 1998; Sharma 2006; Shi et al. 2010). Nested PCR employs two sets of primers in two successive polymerase chain reaction runs in which the first PCR products generated is used as primer for the second PCR (Olsvik et al. 1991). Nested PCR improves the sensitivity and specificity of detecting foodborne pathogens compared to the conventional PCR although the contamination level can be high probably from the laboratory environment since the reaction vessel is opened to enable the second primer set to be added (Picken et al. 1997).

## Molecular techniques for typing pathogens

Several molecular techniques have been developed and extensively used for typing foodborne pathogens. Typing techniques have the advantage that, they allow for the investigation of foodborne outbreaks, give better understanding into the epidemiology of infections and aid in the treatment of infested people (Arbeit 1999; Trindade et al. 2003). Typing techniques are evaluated in terms of their performance (discriminatory power, reproducibility, typeability, and agreement between typing techniques) and convenience in usage (cost and availability of reagents and equipment; rapidity and ease of execution and interpretation of results; and versatility) (Maslow et al. 1993; Struelens 1996; Trindade et al. 2003). Trindade et al. (2003) defined discriminatory power as the probability that isolates sharing identical or intimately related profiles are in fact clonal and part of the same chain of transmission; reproducibility as the ability of a typing technique to yield the same results when the same sample is tested repeatedly; and typeability as the proportion of isolates that can be assigned as belonging to a ‘’type’’ by a typing technique. They also defined the versatility of a typing technique as its ability to type any pathogen with modification of the protocol. Agreement between two typing methods is evaluated by determining if highly similar isolates are grouped accordingly by these techniques (Struelens 1996). Of all these criteria, discriminatory power has been identified to be a key characteristic for typing techniques (Struelens 1996).

Pulsed field gel electrophoresis (PFGE), multilocus sequence typing (MLST), random amplified polymorphism deoxyribonucleic acid (RAPD), plasmid profile analysis, deoxyribonucleic acid (DNA) sequencing are among most often used typing techniques, and have been applied to pathogens isolated from ducks and their environmental sample (Table 3). Others such as repetitive extragenic palindromic (REP), enterobacterial repetitive intergenic consensus (ERIC), ribotyping, amplified fragment length polymorphism (AFLP), and restriction fragment length polymorphism (RFLP) and so on are yet to be reported in terms of their application to duck isolates. Table 3 depicts commonly available molecular techniques that have been applied to type or characterize bacteria isolated from ducks and their related samples; while Table 4 summarizes the advantages and disadvantages of some commonly available molecular techniques for typing or characterizing foodborne pathogens.

**Table 3:** Molecular methods applied to type or characterize bacteria isolated from ducks and their related samples

Typing method	Purpose	References
PFGE	To characterize *S*. Potsdam, *S.* Montevide and *S.* Albany isolated from duck hatcheries	Su et al. (2011)
PFGE	To identify and to characterize *Salmonella* Typhimurium for outbreak investigation	Noble et al. (2012)
MLST	To describe and to compare the genetic diversity of *Campylobacter* colonization in domestic and wild ducks	Colles et al. (2011)
RAPD	To determine the genetic diversity among duck *Campylobacter* isolates	Adzitey et al. (2012a)
Plasmid analysis	To determine the diversity and plasmid size of *Salmonella* serovars	Su et al. (2011)
Plasmid analysis	To identify virulence plasmids in *Salmonella* isolates	Yu et al. (2008)
Plasmid analysis	To determine plasmid size of duck *Salmonella* serovars	Adzitey et al. (2012b)
DNA sequencing	To identify bacteria isolated from duck houses	Martin et al. (2010)
DNA sequencing	To identify bacteria isolated from duck houses and to determine their genetic relatedness	Martin and Jäckel (2011)

**Table 4:** Advantages and disadvantages of some commonly available molecular techniques for typing or characterizing foodborne pathogens

Typing method	Advantages	Disadvantages	References
PFGE^a^	Has high discriminatory power, reproducibility and typeability	Requires 3–5 days to complete a test, the cost is relatively high compared to other methods, this technique has limited availability	Wassenaar and Newell (2000); Trindade et al. (2003)
MLST^a^	Typing data are readily available via the internet and easy to compare results among laboratories and countries, has good discriminatory ability	This method is expensive and will require skilled researcher to perform	Enright and Spratt (1999); Urwin and Maiden (2003); Dingle et al. (2005)
RAPD^a^	Cheap, rapid, readily available and easy to perform	Has average reproducibility, discriminatory power and approximately 80 % typeability	Wassenaar and Newell (2000); Shi et al. (2010)
DNA sequencing^a^	Has high discriminatory power, typeability and reproducibility	Requires more days to complete a test, this method is complex and relatively expensive	Newell et al. (2000); Wassenaar and Newell (2000)
Plasmid analysis^a^	Easy to perform and to interpret the results	Plasmids can readily be lost or acquired and can make genetically related isolates to have different plasmid profiles. This method has poor reproducibility and low discriminatory power compared many typing methods	Hartstein et al. (1995); Trindade et al. (2003)
REP^b^	Cheap, easy to perform and applicable to small or large number of isolates	Discriminatory power, reproducibility and typeability is lower compared to PFGE, MLST and DNA sequencing	Versalovic et al. (1991); Trindade et al. (2003)
ERIC^b^	Quick, cost effective and does not require much skills to perform	Discriminatory power, reproducibility and typeability is lower compared to PFGE, MLST and DNA sequencing	Wassenaar and Newell (2000); Tobes and Ramos (2005)
Ribotyping^b^	Has 100 % typeability, good reproducibility and discriminatory power	It is a complex method and requires 3–4 days to complete a test	Denes et al. (1997); Wassenaar and Newell (2000); Shi et al. (2010)
AFLP^b^	Has good discriminatory power, good reproducibility, 100 % typeability	Requires 3–4 days to complete a test and major capital investment	Wassenaar and Newell (2000); Meudt and Clarke (2007)
RFLP^b^	Inexpensive and very sensitive for strain identification or differentiation	Slow, difficult and could take up to a month to complete	Mohran et al. (1996); Nachamkin et al. (1996); Babalola (2003)

^a^Applied to duck bacterial isolates

^b^Their applications to duck bacterial isolates are unavailable or yet to be published

### Pulsed field gel electrophoresis (PFGE)

Pulsed field gel electrophoresis (PFGE) is an agarose gel electrophoresis technique used for separating larger pieces of DNA by applying electrical current that periodically changes direction (three directions) in a gel matrix unlike the conventional gel electrophoresis where the current flows only in one direction (Schwartz and Cantor 1984; Arbeit 1999; Trindade et al. 2003). In PFGE, intact chromosomes are digested using restriction enzymes or restriction endonucleases to generate series of DNA fragments of different sizes (also known as restriction fragments length polymorphisms, RFLPs) and patterns specific for a particular species or strain (Shi et al. 2010). Pulsed field gel electrophoresis is considered as the ‘gold standard’ typing method by many researchers for foodborne pathogen outbreak investigations and other epidemiological studies (Alonso et al. 2005). This method has good reproducibility, discriminatory power and typeability but PFGE is sensitive to genetic instability, has limited availability and requires at least 3–4 days to complete a test (Wassenaar and Newell 2000). This method is also expensive compared to RAPD, ERIC, REP and plasmid analysis. Degrading of DNA during PFGE process can occur making those strains untypeable, however, this can be resolved (100 % typeability) by modifying PFGE standard procedures (Alonso et al. 2005; CDC 2002). Su et al. (2011) used PFGE to characterize two *Salmonella* Montevideo and 42 *Salmonella* Potsdam isolates from duck hatcheries and found that they belong to the PFGE profile 2, 4 and 5. Comparison of PFGE results revealed that isolates from duck hatcheries were more diverse than those from goose hatcheries (Su et al. 2011). By utilizing PFGE to characterize *Salmonella*, the same strain of Typhimurium DT8 was identified in human and duck egg isolates (Noble et al. 2012). Selected isolates from human, duck eggs, duck meat, duck liver pate and/or dead embryos were indistinguishable using PFGE (Noble et al. 2012).

### Multilocus sequence typing (MLST)

Multilocus sequence typing (MLST) is a an unambiguous, portable and nucleotide-based technique for typing bacteria using the sequences of internal fragments of (usually) seven house-keeping genes (Maiden et al. 1998; Spratt 1999; Urwin and Maiden 2003). In MLST, different sequences within a bacteria species are assigned as distinct alleles for each house-keeping gene and the alleles at each end of the seven loci define the allelic profile or sequence type for each isolate (Urwin and Maiden 2003). Approximately 450–500 bp internal fragments of each gene are used and most bacteria have enough variation within the house-keeping genes to provide many alleles per locus thus allowing billions of distinct allelic profiles to be differentiated utilizing the seven house-keeping loci (Enright and Spratt 1999; Urwin and Maiden 2003). Multilocus sequence typing (MLST) was developed using the concept of multilocus enzyme electrophoresis (MLEE), but instead it assigns alleles at multiple house-keeping genes directly by DNA sequencing (analyses genes themselves) instead of indirectly through electrophoretic mobility (analyses gene expression) for MLEE (Maiden et al. 1998; Spratt 1999; Trindade et al. 2003). The advantages of MLST are that it provides typing data that are unambiguous, portable, more accurate and more discriminatory for most bacteria (Enright and Spratt 1999; Urwin and Maiden 2003). These data are readily available, comparable and accessible via the internet in contrast to most typing procedures involving comparison of DNA fragment sizes on a gel (Enright and Spratt 1999; Urwin and Maiden 2003; Dingle et al. 2005). This makes MLST typing data more suitable for global epidemiological studies. Furthermore, MLST data can be used to investigate evolutionary relationships among bacteria (Urwin and Maiden, 2003). Nonetheless multilocus sequence typing is expensive compared to RAPD, ERIC, REP and plasmid analysis. Due to the sequence conservation in house-keeping genes, MLST sometimes lacks the discriminatory power to differentiate bacterial strains and thus multi-virulence-locus sequence can be used to solve this problem (Chen et al. 2005, 2007). Colles et al. (2011) used MLST to describe and to compare the genetic diversity of *Campylobacter* colonization in domestic and wild mallard ducks.

### Random amplified polymorphism deoxyribonucleic acid (RAPD)

Random amplified polymorphism deoxyribonucleic acid (RAPD) is a PCR-based technique in which arbitrary primers (typically 10-mer primers) are used to randomly amplify segments of target DNA under low-stringency PCR condition (Wassenaar and Newell, 2000). This process leads to the amplification of one or more DNA sequences and generates a set of finger printing patterns of different sizes specific to each strain (Farber 1996, Trindade et al. 2003). The advantages of RAPD are that, it is relatively cheap, rapid, readily available, and easy to perform (Wassenaar and Newell 2000; Shi et al. 2010; Rezk et al. 2012). In RAPD, the efficiency of amplification, annealing and the length of the product varies with the primed sites, giving rise to both weak and strong amplicons which make interpretation of the results difficult (Wassenaar and Newell 2000). In addition, RAPD has low reproducibility, average discriminatory power and approximately 80 % typeability (Wassenaar and Newell 2000). The use of two or more primers improves the discriminatory power of RAPD (Trindade et al. 2003). In a study carried out by Adzitey et al. (2012a) involving the use of RAPD, 94 *C. jejuni* and 19 *C. coli* strains were grouped into 58 and 12 RAPD types, respectively. Analysis of 3 *C. lari* by RAPD also revealed a very high heterogeneity among the isolates. The same researchers have used RAPD to characterize *Salmonella* serovars and *L. monocytogenes* isolated from duck and their environmental samples (unpublished data).

### Plasmid profile analysis

Plasmid profile analysis is one of the oldest molecular techniques used for epidemiological investigation. In this technique, plasmid DNAs are extracted from bacteria and the DNA is separated on agarose gel electrophoresis. It is easy to perform this technique and to interpret the results except that plasmids are mobile extrachromosomal elements that can spontaneously be lost or readily acquired by bacteria and thus isolates that are related epidemiologically can easily display different plasmid profiles (Trindade et al. 2003). The same researchers also reported that plasmids have transposons which may contain resistant determinants that can readily be lost or acquired, quickly changing the composition of plasmid DNA. Plasmids exist in a variety of spatial conformations (linear, nicked and supercoiled) which result in different migration velocities when submitted to agarose gel electrophoresis and this affects the reproducibility of this technique (Hartstein et al. 1995). Su et al. (2011) reported that plasmid analysis of *Salmonella* isolates from duck hatcheries was more diverse than that from goose hatcheries. The isolates (*Salmonella* Montevideo and *Salmonella* Potsdam) belonged to the plasmid profile II (90–50 kb), IV (<6.6 kb), V (90–50 kb; 50–6.6 kb and <6.6 kb) and VI (50–6.6 kb and <6.6 kb) (Su et al. 2011). All *Salmonella* Typhimirium isolated from ducklings (30 ducklings) and duck (1 duck) harboured a 94.7 kb virulence plasmid (Yu et al. 2008). Adzitey et al. (2012b) also reported on the detection of plasmids (ranging from 1.4 to 23.1 kb) in 91 *Salmonella* serovars isolated from ducks and their environment samples in Penang, Malaysia.

### Deoxyribonucleic acid (DNA) sequencing techniques

Deoxyribonucleic acid (DNA) sequencing techniques involve technologies used to determine the order of the nucleotide bases (namely adenine, cytosine, guanine and thymine) in a DNA molecule. In recent times, DNA sequencing is widely and routinely used in the identification, typing, characterization and/or taxonomic classification of unknown or novel pathogens isolates by many researchers. DNA sequencing has always been preceded by PCR to amplify the target genes. 16S rRNA is a common gene that is amplified for sequencing and subsequently for the identification, typing and/or taxonomic classification of the pathogen in question. Sequencing has high discriminatory power, 100 % typeability and good reproducibility (Newell et al. 2000; Wassenaar and Newell 2000). The disadvantage is that, it requires 2–3 days to complete a test, has limited availability and costs higher than other typing methods (Newell et al. 2000; Wassenaar and Newell 2000). In DNA-based methods, different protocols are adapted by different authors and this hampers effective comparison (Newell et al. 2000; Abulreesh et al. 2006). Martin et al. (2010) used 16S rRNA gene sequence analysis to identify bacteria isolates from duck houses at the genus level. The same researchers reported that based on the 16S rRNA gene analyses some isolates were closely related to organisms that may cause pulmonary health effects. 16S rRNA gene analysis was used to identify bacterial isolates from duck hatcheries and these isolates (more than 50 %) were phylogenetically closely related (Martin and Jäckel 2011).

### Other typing methods

These include techniques widely used in typing foodborne pathogens except that their application to pathogens isolated from ducks is yet to be reported. Those briefly reviewed are enterobacterial repetitive intergenic consensus (ERIC); repetitive extragenic palindromic (REP), ribotyping, amplified fragment length polymorphism (AFLP), and restriction fragment length polymorphism (RFLP).

Foodborne bacteria pathogens posses’ sequences of repetitive elements which may be conserved in many genera or species (Lupski and Weinstock 1992; Trindade et al. 2003; Tobes and Ramos 2005). Enterobacterial repetitive intergenic consensus (ERIC) PCR uses primers specific for enterobacterial repetitive intergenic consensus sequences. These primers can be used under high stringency conditions to match the target DNA to produce DNA finger printing that are different in sizes (Wassenaar and Newell 2000; Trindade et al. 2003). Enterobacterial repetitive intergenic consensus (ERIC) PCR is quick, easy to perform and cost effective. Nonetheless, reproducibility is low compared to pulsed field gel electrophoresis (Wassenaar and Newell 2000).

Repetitive extragenic palindromic sequences (REP) also depend on repetitive DNA elements present in foodborne pathogens (Trindade et al. 2003). In repetitive extragenic palindromic, repetitive DNA elements present within bacterial genome are amplified to produce finger printing of different sizes specific to each strain (Versalovic et al. 1991). Trindade et al. (2003) reported that REP is cheaper, easy to perform and applicable to small or large number of isolates and the results have a good correlation with those obtained by PFGE but have lower discriminatory power.

Ribotyping involves the use of selected restriction endonuclease to digest genomic DNA into small DNA fragments which are separated by gel electrophoresis and identified using Southern blot hybridization with a probe specific for rRNA genes (Shi et al. 2010). Ribotyping has 100 % typeability and good reproducibility but it is a complex method, sensitive to genetic instability, and requires 3–4 days to complete a test (Wassenaar and Newell 2000). Ribotyping has higher discriminatory power at the species and subspecies level compared to the strain level (Denes et al. 1997; Shi et al. 2010).

Amplified fragment length polymorphism (AFLP) involves the use of two restriction enzymes to digest total genome DNA, one with an average cutting frequency (4-bp recognition site) and the other with a higher cutting frequency (6-bp recognition site) followed by linking of adapters to the sticky ends of the restriction fragments and amplification of a subset of selected restriction fragments (Wassenaar and Newell 2000; Shi et al. 2010). The primers used for amplification are radioactive or fluorescent labelled and denaturing polyacrylamide gel analysis is used to determine the presence or absence of DNA fragments to identify polymorphisms (Blears et al. 1998; Wassenaar and Newell 2000). Amplified restriction length polymorphism has good discriminatory power, good reproducibility, 100 % typeability, needs no prior sequence information for amplification and insensitive to genetic instability but AFLP is a complex method, requires 3–4 days to complete a test and requires major capital investment (Wassenaar and Newell 2000; Meudt and Clarke 2007).

Restriction fragment length polymorphism (RFLP) involves the use of restriction enzyme to digest DNA and to separate the resulting restriction fragments according to their length on agarose gel electrophoresis. Restriction fragments are then transferred into a membrane through Southern blot procedure and hybridized to a membrane bound labelled DNA probe (Babalola 2003; Foley et al. 2009). This method utilises the variations in homologous DNA sequences to characterize bacteria. This technique is inexpensive, very sensitive for strain identification or differentiation and had widespread application, although it has become obsolete in present times due to the emergence of relatively inexpensive sequencing technologies (Mohran et al. 1996; Babalola 2003). The technology is also slow, difficult and could take up to a month to complete (Mohran et al. 1996; Nachamkin et al. 1996).

## Conclusion

Several detection and typing methods have been developed and are widely used to detect, differentiate, type and/or to classify pathogens for efficient identification, outbreak investigations, clinical treatments and/or epidemiological studies. The combination of two or more primers and/or methods, and optimization of methods will drastically increase the discriminatory power of the detection or typing technique employed. The detection and typing methods reviewed here increase our knowledge on which detection or typing method to go for and the reason for the choice. Studies have also demonstrated that duck eggs, meats or products are important source of foodborne pathogens and have been implicated in a number of foodborne outbreaks. Nonetheless, limited surveillance studies are available as far as the isolation of foodborne pathogens in ducks and their related samples are concern. This has reflected in the relatively very low available literature on the application of molecular techniques to detect or type foodborne pathogens isolated from ducks.
